# High efficacy vasopermeability drug candidates identified by screening in an *ex ovo* chorioallantoic membrane model

**DOI:** 10.1038/srep15756

**Published:** 2015-10-29

**Authors:** Desmond Pink, Keith A. Luhrs, Longen Zhou, Wendy Schulte, Jennifer Chase, Christian Frosch, Udo Haberl, Van Nguyen, Aparna I. Roy, John D. Lewis, Andries Zijlstra, Missag H. Parseghian

**Affiliations:** 1 Innovascreen Inc., 1959 Upper Water St. Suite 1700, Halifax, NS, B3J 3N2, Canada; 2 University of Alberta, 114th Street & 87th Avenue, Edmonton, AB, T6G 2E1, Canada; 3 Peregrine Pharmaceuticals Inc., 14282 Franklin Avenue Tustin, CA, 92780, USA; 4 BioBroker, Arnold-Sommerfeld-Ring 2, 52499 Baesweiler, Germany; 5 Vanderbilt University Medical Center, 1161 21st Ave. S., Nashville, TN, 37232, USA

## Abstract

The use of rodent models to evaluate efficacy during testing is accompanied by significant economic and regulatory hurdles which compound the costs of screening for promising drug candidates. Vasopermeation Enhancement Agents (VEAs) are a new class of biologics that are designed to increase the uptake of cancer therapeutics at the tumor site by modifying vascular permeability in the tumor to increase the therapeutic index of co-administered drugs. To evaluate the efficacy of a panel of VEA clinical candidates, we compared the rodent Miles assay to an equivalent assay in the *ex ovo* chicken embryo model. Both model systems identified the same candidate (PVL 10) as the most active promoter of vasopermeation in non-tumor tissues. An *ex ovo* chicken embryo system was utilized to test each candidate VEA in two human tumor models at a range of concentrations. Vasopermeation activity due to VEA was dependent on tumor type, with HEp3 tumors displaying higher levels of vasopermeation than MDA-MB-435. One candidate (PVL 10) proved optimal for HEp3 tumors and another (PVL 2) for MDA-MB-435. The use of the *ex ovo* chicken embryo model provides a rapid and less costly alternative to the use of rodent models for preclinical screening of drug candidates.

For years, the chorioallantoic membrane (CAM) of the avian embryo has been exploited in the study of angiogenesis and tumor cell metastasis. More recently, an *ex ovo* adaptation of this system has been exploited in the study of vascular permeability and vascular leakage^1^. Prior to the use of chicken embryos, studying the separate molecular processes of vascular permeability and leakage required artificial *in vitro* assays (e.g. Boyden chamber) or expensive *in vivo* analysis of Evans Blue dye extravasation in rodent tissues^2^. The *ex ovo* CAM is a system that combines the versatility of *in vitro* assays with the tissue complexity of higher order *in vivo* systems. A highly vascularized extra-embryonic membrane connected to the embryo through a continuous circulatory system, the CAM is readily accessible for experimental manipulation, including the intravenous injection of candidate drugs and the direct visualization of local responses. Having recently demonstrated the usefulness of this model to evaluate the impact of vasopermeation on drug uptake in tumors^1^, we elected to utilize this system to screen a class of drugs known as Vasopermeation Enhancement Agents (VEAs). These agents consist of tumor-specific antibodies fused to vasoactive compounds that are designed to induce vascular permeability at the tumor site^3^. In particular, we focus on a VEA that had been chosen for clinical development that uses the NHS76 antibody fused to Interleukin 2 (IL-2). NHS76 is a fully human antibody that binds the histone/DNA complex normally found within the nucleus, however, it is also capable of targeting the complex when it is exposed extracellularly in regions of tissue necrosis, such as occurs in the core of a solid tumor^4^. The use of systemic IL-2 once held great promise as a cancer therapy^5^, however, its role in causing vascular leak syndrome (VLS), which results in interstitial edema and organ failure, has limited its usage^6^. IL-2 residues responsible for vascular leakage and toxicity have been identified at residues D20^7^ and N88^8^. However, the phenomenon of vascular leakage appears to be wholly separate from vascular permeability or vasopermeation caused by IL-2 as evidenced by mutation of the R38 residue^9^ located within a region bounded by residues Q22 to C58 and encompassing the linker region between α-helices A and B as well as parts of the helices themselves (Fig. 1A; fragment within the IL-2 protein structure marked in red). Isolation of this IL-2 fragment has been shown to still possess vasopermeation activity^1^,^10^ which can be directed to the tumor microenvironment when fused to the NHS76 antibody^11^. This fragment is now referred to as the permeability enhancing peptide, PEP^10^, and is located within the mature IL-2 protein structure in a region expected to interact with IL-2 receptors α and γ [IL-2Rα, IL-2Rγ^12^]. The PEP fragment includes the highly conserved RMLTFKFY amino acid sequence known to interact with IL-2Rα^13^,^14^,^15^ and lacks cytokine activity^10^. While the ability of the PEP fragment to induce vasopermeability has been documented in the literature, the mechanism of action is still unclear and whether vasopermeation is caused when PEP interacts with IL-2 receptors or some other receptors is unknown. Due to the complexity of vasopermeability responses, efficacy testing of the compound must necessarily be conducted in animal tumor models, which poses challenges for the eventual development of a validated potency assay.

Here we present the results of a study evaluating a panel of five human IL-2 deletion variants fused to NHS76 in order to identify a structurally stable molecule that has the greatest vasopermeation activity. The potency of each candidate was evaluated in the rodent Miles assay and compared to a modified Miles assay^2^ adapted to the *ex ovo* chicken embryo model. Vasopermeation activity was assessed in both normal and tumor vasculature. To assess vasopermeation changes in the tumor microenvironment, two human xenograft tumor models (HEp3 epithelial carcinoma and MDA-MB-435 breast cancer) were evaluated. A fluorescence intravital imaging approach was employed to visualize the dynamics of vasopermeation activity in these tumor models.

## Results

### Generation of a panel of targeted vasopermeation enhancement agents (VEA)

**Figure 1:**
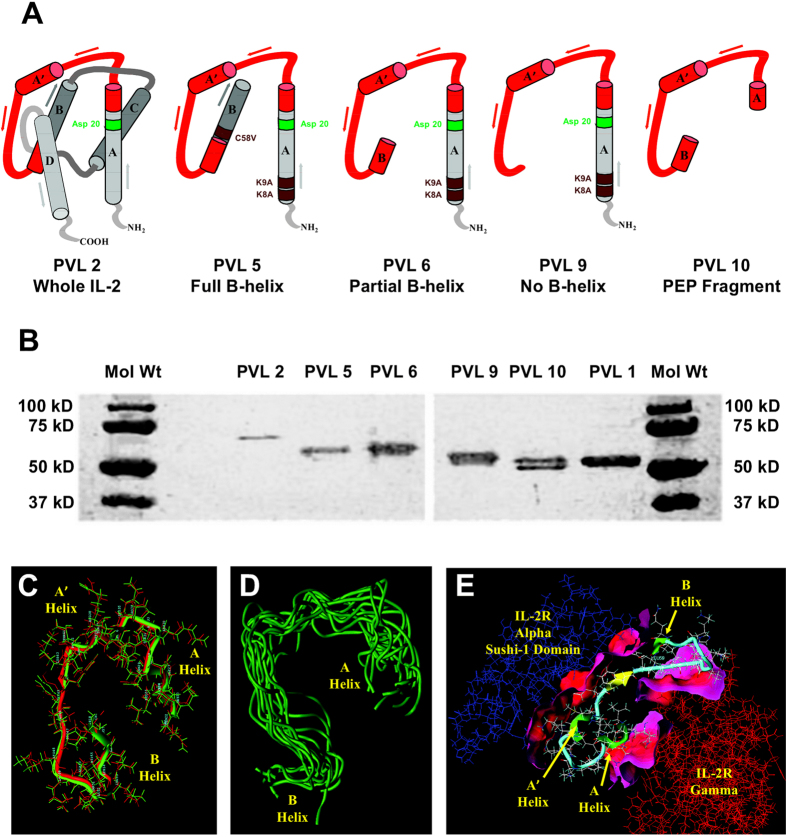
Generation of a panel of targeted VEAs. (**A**) Schematic representation of IL-2 and its deletion mutants which were fused to the C-terminal tail of the NHS76 heavy chain to create the VEA candidates being tested. The A, A′, B, C and D helices making up the secondary structure of IL-2 are represented by cylinders. The PEP region is colored red. Aspartic acid at residue 20 (Asp 20 or D20) is part of the “xDy motif” known to cause vascular leak^7^. Point mutations converting lysines to alanine (K8A, K9A) and cysteine to valine (C58V) improve product stability. (**B**) Purified PVLs were analyzed by SDS-PAGE on a 4–20% gradient gel. Samples were reduced with β-mercaptoethanol, heated to 95 °C for 5 minutes before being resolved into heavy and light chain bands and visualized with Coomassie stain. The heavy chain bands are shown here to highlight differences in migration patterns for each construct and their correlation to the estimated molecular weights in Table 1. The lanes marked Mol Wt contain proteins of known molecular weight and illustrate their migration pattern in this gradient gel. (**C**) Energy minimization analysis using SYBYL *Dynamics* software compares the expected PEP monomer structure in solution (green) with the homologous peptide fragment comprising the native IL-2 sequence (red) and finds the two structures nearly identical. (**D**) Overlay of the minimized structures of the PEP fragment, after simulated annealing from 700 K down to 0 K, illustrated with single green tubes representing each of the energy minimized PEP backbones after various annealing cycles. Note the overall similarity of the low energy conformations for PEP throughout the 50 simulated annealing cycles. (**E**) Interaction analysis of PEP fragment (light blue) with the IL-2 α receptor (blue) and IL-2 γ receptor (red) using SYBYL *Biopolymer, Tripos Force Field* and the *Powell minimization* algorithm. The separating surface (rendered by MOLCAD) between the PEP molecule and the individual receptor chains represent the interaction surface between molecules in contact with one another. The color gradient from red to pink represents the distance between interacting molecules with the closer proximity represented in deeper shades of red. Only a portion of the IL-2 α receptor is represented in the image.

To generate a panel of novel targeted VEAs, deletion mutants of IL-2 were fused to a variant of the NHS76 antibody and screened to identify the shortest structurally stable IL-2 fragment with optimal vasopermeation activity. Figure 1A illustrates five of the IL-2 mutants studied (Table 1), all of them containing the PEP fragment (in red) and four of them also including the D20 site responsible for vascular leak syndrome^7^. The PEP region ends at residue C58^10^. To reduce the complication of disulfide bond formation during product storage, PVLs 6, and 10 were truncated at residue Q57, while PVL 5 had its C58 residue converted to V58. Finally, deletion mutations of IL-2 expose a plasmin cleavage site identified between lysine residues 8 and 9 in the mature IL-2 primary sequence [Stephen Gillies, personal communication]. The tandem lysines at this site were mutated to alanines in order to improve serum stability (PVLs 5, 6 and 9). The NHS76 in these studies was a modified IgG2 with the N297 glycosylation site mutated to Q297 to eliminate post-translational carbohydrate modification of the antibody. The IgG2 constant region is known to have an increased *in vivo* circulating half-life compared to its IgG1 counterpart^16^. The DNA sequence for each IL-2 construct was fused to a modified C-terminus of NHS76 with the IgG terminal lysine residue converted to alanine, which was also designed to reduce serum cleavage and improve circulating half-life^16^. The PVL DNA constructs were then transfected into CHO cells for production. As proof our constructs were expressing the described fusion proteins, the heavy chain from each construct and the NHS76 IgG2 control (PVL1) were resolved using gel electrophoresis (Fig. 1B). The NHS76 portion of the molecules were evaluated for their binding to a mixture of histones and DNA complexed in a 5:1 ratio and adsorbed to the surface of a 96-well plate. Potency binding curves were generated by comparing the binding of the purified PVLs to the PVL1 (acting as a reference standard), in triplicate, at several concentrations. The signal generated at each concentration was plotted as a sigmoidal dose-response curve and the linear portion of the curves from the sample and control were compared. At three concentrations in the linear region of both curves, an analysis of variance (ANOVA) algorithm was used to calculate the relative potency of each PVL sample to PVL1 and this value is presented as a percentage of potency (see Binding Potency in Table 1). While the mechanism of vasopermeation is unclear, determining the activity of the IL-2 portion of the PVL constructs using a standard T-cell proliferation assay would be uninformative, particularly since the PEP fragment has no IL-2 cytokine activity^10^. Instead, we tested the vasopermeation activity of each molecule using non-tumor bearing CAMs (see below).

To determine if the VEAs themselves are toxic in the avian system, 1.5 nmoles of reagent (approximately 240 μg) was injected into the CAM of 15 day old embryos and mortality assessed at 24, 48 and 72 hours (i.e. days 16, 17 and 18). CAMs injected with each of the PVLs (n = 5 each) had an equivalent mortality to those injected with 50 μL PBS (n = 40) between days 15 and 18 (Table 2), indicating a lack of toxicity. None of the endotoxin levels were sufficient to cause an increase in toxicity above background nor was there a correlation of embryo survival to endotoxin levels. As a positive control, a cohort was injected with 50 μL of lipopolysaccharide (LPS; 1000 EU/mL) with all five embryos dying within one hour (Table 2). PVLs 5, 6 and 9 were chosen as VEA candidates under the assumption that all or part of the A and B helices are necessary for the 3-dimensional structure of the PEP fragment. PVL 10 significantly differs in concept from the others, reducing the effector portion of the VEA only to the PEP sequence of amino acids under the assumption that its secondary structure within the intact IL-2 molecule is not critical to its functionality. Computer modeling of PEP provided a surprising result. A native structure for human IL-2 was downloaded from the Protein Data Bank (model 1ILM deposited by^17^), along with the auxiliary IL-2 structural data in PDB entry 3INK (http://www.rcsb.org/pdb/explore/explore.do?structureId=3INK), and further optimized using the Tripos SYBYL software suite. Figure 1C shows the results of an energy minimization analysis of the PEP sequence both within an intact IL-2 molecule (red) and as a free peptide unencumbered by the remaining IL-2 structure (green). The structures are nearly identical, including the presence of the A′ helix located within the PEP fragment. Further analysis of the PEP folding behavior by the simulated annealing method, using the SYBYL *Dynamics* module, calculated structural conformations of PEP as the fragment was heated up to 700 K and then annealed to 0 K and minimized in 50 cycles. Figure 1D illustrates that the majority of the resulting low energy structures assume an almost identical conformation compared to the original PEP structure embedded within the intact IL-2 molecule. Therefore, it is highly likely that the PEP structure attached to the C-terminus of the NHS76 antibody in PVL 10 assumes a conformation that is similar to PEP as part of an intact IL-2. Computer modeling of the PEP fragment in complex with the IL-2 receptors shows an extensive interface with both the IL-2Rα’s Sushi-1 domain and IL-2Rγ even when much of the surrounding IL-2 structure has been removed (Fig. 1E). While this gave us the confidence that a PEP fragment *sans* intact IL-2 could interact with the IL-2 receptors alone, it was not clear whether steric hindrance would disrupt the binding upon fusion to NHS76, nor could it be ruled out that PEP interaction with another receptor may be the cause of vasopermeation.

### Dose optimization studies

**Figure 2:**
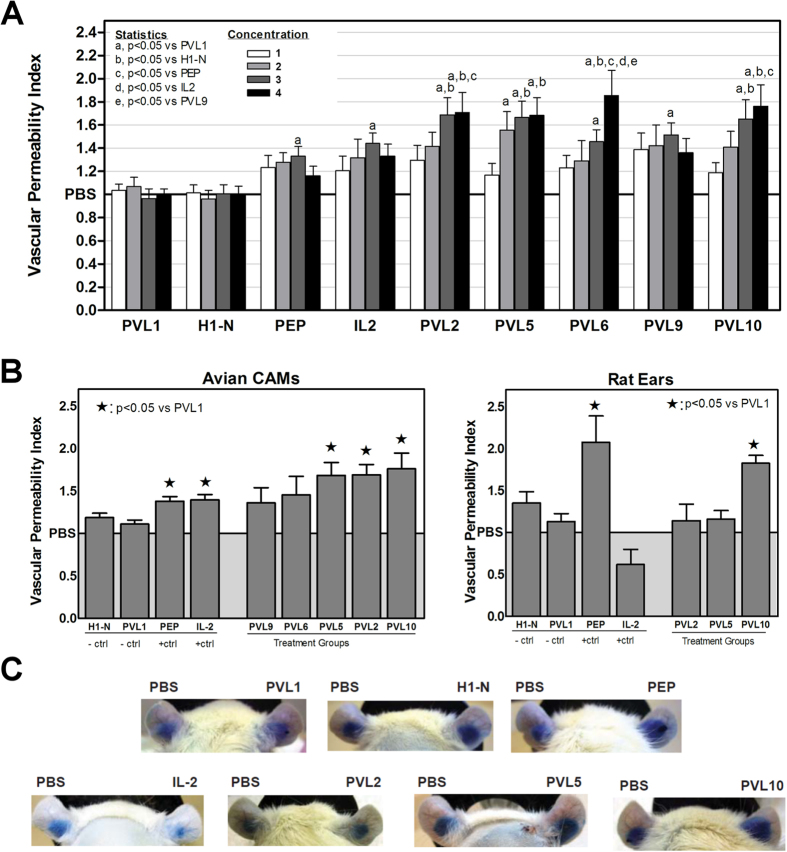
Vascular permeability analyses in non-tumor bearing tissue. (**A**) VPIs for control and experimental reagents in non-tumor bearing CAMs represent the average value from 5 or 6 experiments run in triplicate (*n* = 15–18) at each concentration. Concentrations for control reagents are (1) 0.001 nM, (2) 0.01 nM, (3) 0.1 nM and (4) 1 nM; Concentrations for experimental reagents are (1) 0.005 nM, (2) 0.05 nM, (3) 0.1 nM and (4) 0.15 nM, (**B**) Comparison of average VPIs obtained with each control and VEA candidate under similar conditions in non-tumor bearing CAMs and rats using optimal concentrations determined in A. Average VPI values for the avian CAMs was determined from (PVL1, n = 56; H1-N, n = 41; PEP, n = 44; IL-2, n = 56; PVLs, 15–18). Average VPI values for the rat ears was determined from (PVL1, n = 5; H1-N, n = 3; PEP, n = 5; IL-2, n = 4; PVL2, n = 3; PVL5, n = 3; PVL10 n = 7). (**C**) Representative images for the controls and PVLs tested in the rat Miles assay. Ears were photographed prior to euthanasia and tissue processing.

To find the appropriate dose range for these agents, each was injected intravenously into 15 day chicken embryos at 4 different concentrations (0.001 nM, 0.01 nM, 0.1 nM or 1.0 nM) as previously described^1^. Embryos were then incubated for 2 hours prior to injection of a 0.5% Evan’s Blue dye and further incubation for 1 hour. 1 cm^2^ of tissue was surgically excised, rinsed in PBS, weighed and then homogenized in formamide prior to incubation at 37 °C for 48 hours. After pelleting tissue debris, dye extravasation was quantified spectrophotometrically (λ = 620 nm), the value subtracted from a formamide blank and divided by the weight of the tissue. The NHS76 IgG2 variant (PVL 1) was used as a negative control because it lacked any IL-2 fragment. A peptide control codenamed H1-N, the first 35 amino acids from a histone H1 subtype (H1^S^-3^18^), was also used given its similarity in size to the 37 residue PEP fragment. Positive controls were the PEP fragment and human IL-2. A vascular permeability index (VPI) was calculated as a ratio of the dye concentration per weight of reagent treated tissue divided by the dye concentration in matched vehicle (PBS) treated samples. This ratio indicates little to no vascular permeability when the value is close to 1, as is the case for PVL 1 and H1-N (Fig. 2A). In contrast, both PEP and IL-2 stimulated robust extravasation of dye that was measurable using the modified chicken embryo chromogenic assay. The experimental VEA reagents were also injected into chicken embryos and their VPIs determined at each of four concentrations (Fig. 2A). The optimal VPIs for the VEAs and controls were obtained either at 0.1 nM (PVL1, H1-N, PEP, IL-2, PVLs 2 and 6) or 0.15 nM (PVLs 5, 9 and 10) as illustrated in Fig. 2B (left panel).

### Avian embryo vasopermeation assays produce similar results to the rat ear assay

The VPI for the VEA and control reagents were assayed using rat ears, which are highly vascularized and easily manipulated. Cost and vivarium constraints limited our study to the three most promising VEA candidates in the CAM study (PVLs 2, 5 and 10). Ten milliliters of Evans Blue dye per kg body weight (0.5% Evans blue (w/v) in endotoxin-free PBS) was injected into the rats. Ten minutes later, the rats were injected intradermally with 25 μL of PBS into the left ear and 0.15 nmoles of reagent (~25 uL) into the right ear. Thirty minutes after injection of reagent and PBS, rats were anesthetized, their ears photographed, and then the animals were perfused with 100 mL PBS thru a ventricular infusion followed by ear tissue processing and data collection as described previously^1^. The VPI was calculated by comparing the weight-normalized spectrophotometric data (λ = 620 nm) from each right ear to the contralateral PBS injected left ear. As expected, the PVL 1 control showed no significant vasoactivity while the PEP control did. Unexpectedly, the human IL-2 provided a result <1, suggesting suppression of background vasoactivity normally seen during a PBS injection (Fig. 2B right panel and 2C). The result is partially explained by the fact that extravasation is allowed for 30 minutes after injection of the test reagent followed by harvesting of the ears. Careful observation after injection of human IL-2 repeatedly showed extravasation of Evans Blue dye in a matter of minutes followed by rapid dissipation such that there appeared to be less vasoactivity in the IL-2 injected ear at 30 minutes than the contralateral PBS injected one (Fig. 2C). While the mechanism for this phenomenon is not clear, each of the rats was treated with care not to pierce any adjacent blood vessels. This phenomenon may explain why PVL 2, containing two intact IL-2s at the C-terminus of each NHS76 heavy chain, and PVL 5, similarly containing a large IL-2 fragment, showed no significant vasoactivity compared to PVL 1. Conversely, PVL 10 proved quite vasoactive both in the rat and chicken models (Fig. 2B).

### Comparison of VEA activity in two tumor models

**Figure 3:**
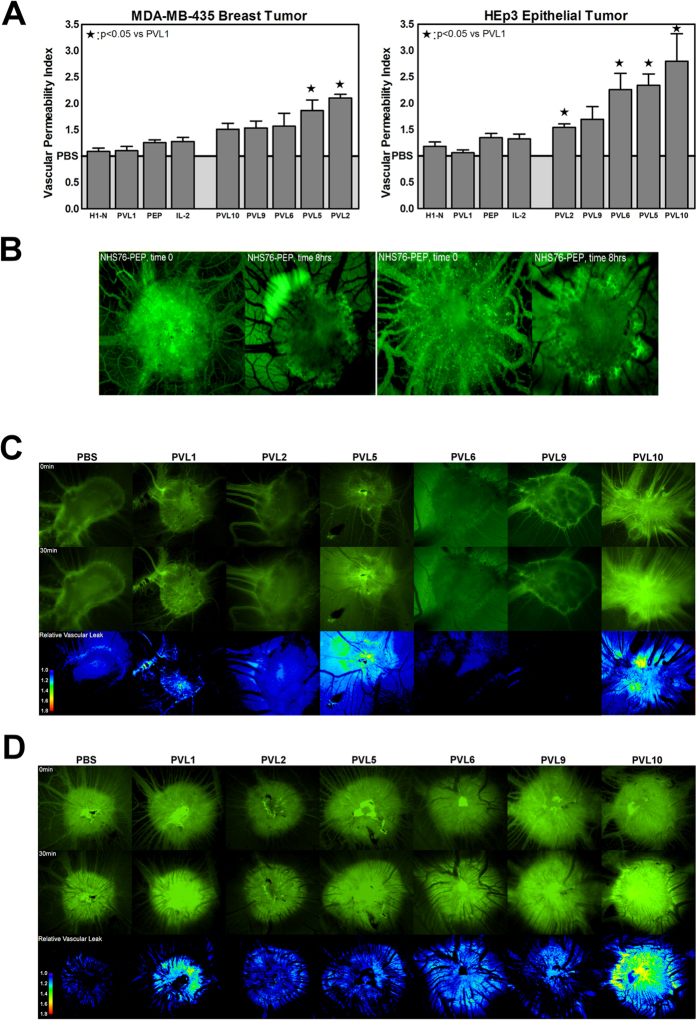
Vascular permeability analyses in tumor-bearing tissue. (**A**) Graphical comparison of average VPIs obtained with each control and VEA candidate in tumor bearing CAMs. Representative VPIs for the PVLs and controls were chosen based on normalized values. For the MDA-MB-435 data set, PVL1, H1-N, PEP, IL-2, PVLs 2 and 6 had VPIs from the 0.1 nM concentration data and PVLs 5, 9 and 10 from the 0.15 nM data. For the HEp3 data set, PVLs 2, 5, 6 and 10 had VPIs from the 0.15 nM data set, PVL1, H1-N, PEP and IL-2 from the 0.1 nM data set and PVL 9 from the 0.05 nM concentration data set. (**B**) HEp3 cells were cultured on the CAM until a necrotic core was established. Then NHS76-PEP was injected at a site distant from the tumor and the embryos incubated for 1 to 8 hours prior to the addition of FITC-dextran to track the flow of blood plasma into the tumor’s interstitial spaces. Over an 8 hour time lapse, there is progressive extravasation of dextran and, thus, blood plasma at the site of the tumor. Pictures shown are two separate experiments. Note the presence of fluorescein in the vessels surrounding the tumor at time 0, but its near total absence from the vessels at 8 hours. (**C**,**D**) Qualitative assessment of the relative vascular leak after 30 min for each PVL candidate tested in MDA-MB-435 and HEp3 bearing CAMs. Sample images for PBS, PVL1, PVL2, PVL5, PVL6, PVL9 and PVL10 candidates are shown for both (**C**) MDA-MB-435 tumor bearing CAMS and (**D**) HEp3 tumor bearing CAMS. Images were aligned (Stack Reg plugin, Rigid translation in Image J (1.47v) and the relative change in vascular leak was visualized by subtracting the 0 min time point image from the 30 min time point image using the Image calculator function.

To evaluate the activity of these experimental VEA candidates in human tumors, two xenograft tumor cell lines, HEp3 epithelial carcinoma and MDA-MB-435 breast cancer, were used. These cell lines were chosen due to their dramatically different tumor architecture. Tumors comprised from HEp3 cells are fast-growing and highly vascularized, with little to no necrotic areas. In contrast, MDA-MB-435 tumors develop a rapidly-proliferating periphery of well-vascularized tissue surrounding a poorly-vascularized necrotic core. We were interested in evaluating the relative effect of NHS76 targeting on these distinct tumor architectures. The effect on tumor-bearing CAMs was measured quantitatively to determine the VPI for each drug candidate in each cancer cell line. Similar to the tumor-free CAMs, the PVL-1 and H1-N negative controls did not increase vasopermeation in MDA-MB-435 or HEp3 tumors (Fig. 3A). Vascular permeability was compared between non-tumor and tumor tissues to assess whether activity was tumor-targeted. No significant differences in vasopermeability were observed for any of the controls (PVL1, H1-N, PEP or IL-2) when comparing tumor bearing to the non-tumor bearing embryos (2-way ANOVA (Bonferonni post-test). Injection of PEP or IL-2 as positive controls stimulated vasopermeation in the MDA-MB-435 bearing CAMs in a dose-dependent manner with a maximum activity at 0.1 nM (Fig. 3A, left panel; Supplementary Fig. 1), similar to that seen in non-tumor bearing CAMs (Fig. 2A). Control agents had equivalent responses in both tumor types (Fig. 3A). PVL 2, which consists of the intact IL-2 fused to NHS76, exhibited greater activity in MDA-MB-435 tumors than the other candidates. For the highly vascularized HEp3 tumors, PVL 10 exhibited greater activity than the other candidates (Supplementary Fig. 1; Fig. 3A, right panel).

### Fluorescence time-lapse imaging of tumor vascular permeability

The transparent CAM permits real-time microscopic observation of blood flow after intravenous injection of fluorescent dyes such as 70 kD fluorescein dextran. The immunocompromised status of early chick embryos^19^ allows for the cultivation of xenografted tumors in the CAM^20^.

Using an epifluorescence microscope, the effects of several VEA candidates were screened in CAMs implanted on day 11 either with the HEp3 epithelial carcinoma or MDA-MB-435 breast cancer line. Both tumor types show significant vascularization after 3 days growth, and the MDA-MB-435 tumors develop a necrotic core after 7 days. Using a fluorescence intravital imaging approach, the impact of intravenously injected NHS76-PEP (PVL 10) on the leakage of 70 kD FITC-dextran in HEp3 tumors was evaluated over 8 hours. Over the 8 hour time lapse, a progressive extravasation of the injected dextran, and thus blood plasma, is observed into the interstitial spaces of the tumor (Fig. 3B). In fact, significant differences in the vasopermeability of the 70 kD FITC-dextran were observed after only 30 minutes. Given the large number of CAMs being evaluated in this study, and the relatively rapid timeframe to see significant leakage, intravital imaging studies were conducted using a 30 minute timeframe in CAMs bearing either MDA-MB-435 (Fig. 3C) or HEp3 tumors (Fig. 3D). In those studies, PVLs or control reagents were injected intravenously, the embryos incubated at 37 °C for 2 hours and then a mixture of FITC-dextran (to visualize blood flow), rhodamine-labeled *Lens culinaris* agglutinin-A (LCA) lectin (to visualize blood vessels) and Hoechst 33342 (to visualize endothelial and red blood cells) were injected and fluorescent images collected at times 0, 15 and 30 minutes (Supplementary Information). They reveal no red blood cells outside the vasculature in the vicinity of the tumors during the 30 minute observation period; a strong indication that vasopermeation of blood plasma is not accompanied by significant hemorrhaging. The rhodamine-LCA staining (red) reveals HEp3 tumors are much more vascularized compared to their MDA-MB-435 counterparts. The representative images shown in Fig. 3C,D indicate that the observed leakage in these intravital imaging experiments reflect what is seen in the quantitative vasopermeation assay, and may provide an additional method to assess the efficacy of novel VEA formulations.

## Discussion

The tumor microenvironment is characterized by high interstitial fluid pressure which acts as a barrier limiting the penetration of chemotherapeutics to a fraction of the dosage provided to a patient, hence, lowering the effectiveness of current treatments^21^. And while the conditions responsible for this phenomenon are varied^22^, so are the strategies to overcome it, including inhibition of the PDGF receptor using imatinib to decrease stromal cell contraction^23^, increasing the blood pressure within the tumor using the vasoconstrictor angiotensin II^24^, remodeling of the extracellular matrix using collagenase and hyaluronidase^25^,^26^, normalizing the vascular structure within tumors using anti-VEGF antibodies^27^,^28^, and increasing vasopermeation using the PEP fragment of interleukin-2^11^.

Vasopermeation is a characteristic of IL-2 that appears to be distinct from vascular leakage^9^,^10^. It has shown promise as a way to improve the therapeutic index of co-administered chemotherapies when harnessed to a targeting antibody and localized to the tumor microenvironment^11^; however, it is not clear whether the mechanism of action is predicated on PEP binding the IL-2 receptors or a receptor yet to be identified. Indeed, demonstration of PEP vasoactivity in CAMs, which lack a mature immune system, combined with the observation that this fragment has no IL-2 cytokine activity^10^, prevents us from ruling out the existence of a novel receptor for PEP. *In silico* structural studies were conducted to identify candidates for screening based on the theory that vasopermeation occurs when PEP interacts with IL-2 receptors α and γ (Fig. 1E). It was surprising to find out that PEP’s “random” coil structure within the intact IL-2 is structurally stable even when the fragment is in solution (Fig. 1C,D) so the molecule will likely have the same structure regardless of whether it interacts with IL-2R α and γ or other receptors.

The financial risk of screening a variety of VEA candidates in a costly animal system was reduced by conducting the first round of studies *ex ovo*. To evaluate 5 potential VEA candidates consisting of an antibody (NHS76) fused to an IL-2 fragment, we used 1848 chicken embryos. Of these, 552 were used for the controls. In comparison, we were limited to using 30 rats for the Miles assays. *Ex ovo* evaluation of drug candidates at the first step in the pre-clinical evaluation process allowed us to statistically power the results by using a large number of eggs without having to be hampered by vivarium costs. The capacity of 37 °C incubators to maintain the embryos and the manpower to screen the CAMs with a microscope were the principal resources needed. The ability to grow tumors on the CAMs^20^ allowed for easy visualization of the processes within the microenvironment before and during treatment without the need for surgical manipulation, as is the case in rodent models. Here, we presented a time lapse visualization of increased vasopermeation after VEA administration using fluorescent markers (Fig. 3B–D, Supplementary Information), however, the markers could be easily replaced with actual chemotherapies to visualize the improvement VEAs may provide co-administered drugs [see Fig. 4 in^1^ for a non-VEA example]. Without the use of radioactive or infrared labels and expensive imaging systems, a rodent model generally relegates the researcher to measuring the size of tumor shrinkage as a confirmation of efficacy^11^.

Our comparison of the avian and mammalian screening systems both identified PVL 10 as the most promising candidate for enhancing vasopermeation in non-tumor tissues. Both models also verified that PEP vasoactivity is not lost, due to steric hindrance, with its fusion to an antibody. However, differences were seen with other reagents tested in the two systems. The contradiction in observations between human IL-2 and its PEP fragment in rats may be explained by the presence of the xDy motif at residue D20, known to cause vascular leak syndrome. The presence of residue D20 in PVLs 2 and 5 may also explain differences in their VPI values between the two assay systems (Fig. 2B).This highlights the fact that the mammalian rat model has an intact immune system while CAMs are not fully immunocompetent^19^. The immunocompromised status of the avian embryo allows for the culturing of tumors and the testing of new drugs, as illustrated here with the MDA-MB-435 breast tumor and the highly vascularized HEp3 epithelial carcinoma. In this study, two tumors with contrasting microenvironments were used in the screening process, and significant performance differences were revealed. Besides being highly vascularized, HEp3 cells form spherical tumors (Fig. 3D). In contrast, MDA-MB-435 tumors are more highly variable in shape and have larger areas of necrosis with angiogenesis primarily occurring at the edges of the microenvironment (Fig. 3C). To account for increased background vasoactivity expected in HEp3 tumors compared to MDA-MB-435s, the average VPIs obtained for each tumor type were divided by the average VPI obtained in non-tumor tissue to represent normalized vasopermeation resulting from each PVL or control reagent tested. Plotting the VPIs for each PVL from the drug concentration reveals PVL 2 improved the vascular permeability of the breast tumor, whereas, PVL 10 increased it in the epithelial carcinoma (Fig. 3A,C,D). While the reason for this difference remains to be elucidated, it provides the developer with the option of choosing the best drug candidate for a specific cancer or, if the candidate has been chosen based on other criteria, the best clinical indication suitable for the drug. All before going to the subsequent expense of running mouse models.

## Methods and Materials

### Reagents

All VEAs with the PVL designation were produced at Peregrine Pharmaceuticals, Inc. (Tustin, CA). PEP was synthesized at the University of Southern California Microchemical Core Facility (Los Angeles, CA). H1-N was synthesized by Missag H. Parseghian at the University of California, Irvine. Human IL-2 was purchased from PeproTech (Cat. # 200–02; Rocky Hill, NJ). Hoechst 33342 and fluorescein isothiocyanate (FITC)-dextran (70 kDa) were purchased from Sigma (St. Louis, MO). Rhodamine labeled *Lens culinaris* agglutinin-A (Vector Laboratories, Burlington, ON).

### PVL Variant Creation and Protein Expression

The NHS76 IgG2 fusion molecules to IL-2 or its fragments were synthesized based on the human IL-2 sequences published in UniProt (protein sequence entry: P60568) and GenBank (nucleotide sequence entry: J00264.1). Sequences were synthesized at Blue Heron and ligated into the NheI/BamHI (sticky/blunt) sites of pcDNA™ 5/FRT plasmids before being amplified in *E. coli* and then transfected into Flp-In™ CHO cells (both plasmids and CHO cells from Life Technologies, Grand Island, NY). The Flip-In™ cell lines contain a single integrated FRT site at a proprietary transcriptionally active locus in the CHO genome. When an FRT expression plasmid containing the PVL construct is transfected along with a similar vector carrying the Flp recombinase, there is rapid integration of genes into a single locus, thus resulting in isogenic and stable CHO cells expressing the PVL. We initially transfected an entire T-150 of 90–95% confluent Flp-In CHO cells. These were then split into 5 T-150s each (keeping all of the cells) for selection with 800 μg/ml hygromycin. A high concentration of hygromycin allowed us to select for transfected cells. The cells were grown in Ham’s F-12 with serum before and after transfection, as well as through the selection phase. After about 1.5–2 weeks of selection in hygromycin, depending on how the colonies grew out, cells were transferred into 125 ml spinner flasks and grown in serum-free media CHO CD (Irvine Scientific, Santa Ana, CA). Spinner cultures were started between 2 × 10^5^–5 × 10^5^ cells/ml with the hygromycin concentration reduced down to 150 μg/ml. These cells were kept between 3 × 10^5^ and 1 × 10^6^ cells/ml for 1.5–2 weeks to adapt to the serum free conditions. Cells were then scaled up to 500 mL and then into 2L cultures containing CHO CD media, 100 μg/ml hygromycin, 1x HT (hypoxanthine/thymidine) supplement and 8 mM Glutamax (both Life Technologies). Antibody production was tested with an ELISA sandwich assay using an anti-human IgG to capture the NHS76 and its fusion variants.

### Cell Lines and Tumor Xenografts

HEp3 and MDA435 cells were maintained in Dulbecco’s Modified Eagle Medium (DMEM) supplemented with penicillin/streptomycin and 10% FBS and cultured at 37 °C in 5% CO2 incubator and passaged every 2 to 4 days. Fertilized Dekalb White chicken eggs were incubated in a humidified chamber at 37 °C and on day 4 embryos were removed from their shells using a Dremel tool with a cutting wheel. Embryos were maintained under shell-less conditions, in a covered dish in a humidified air incubator at 37 °C and 60% humidity. For studies involving tumors, day 10 chicken embryos had 0.1–0.5 × 106 epidermoid carcinoma (HEp3) or breast tumor (MDA-MB-435) cells in serum free media applied directly to a section of the CAM surface that had been lightly abraded with a piece of filter paper. Sterilized coverslips were applied on top of the tumor 24 hr post-tumor cell application for embryos being imaged with epifluorescence microscopy. All animals were housed, maintained and treated by procedures approved by and in accordance with University of Alberta Institutional Animal Care and Use Committee (IACUC).

### Quantitative detection of vasoactivity

Vasopermeation was detected in the modifed Miles assay format described for CAMs and rat ears in^1^. One-way ANOVA with Tukey’s post test was performed using GraphPad Prism version 5.04 for Windows, GraphPad Software, San Diego California USA, www.graphpad.com. Data are presented as Mean ± SEM; ★ represents a significant difference, p < 0.05 compared to PVL1.

### Microscopy and Intravital Imaging of CAMs

Tumor cells were placed on the CAM of individual chicken embryos on day 11, as described above, and allowed to grow. Test reagents were injected intravenously on day 18 (0.1 nM for controls and 0.15 nM for PVLs). Each reagent was introduced to three CAMs. Embryos were then incubated for 2 hours at 37 °C before being intravenously injected with a 60 μL cocktail of 1 mg/mL (FITC)-dextran (70 kDa), 0.5 mg/mL rhodamine-LCA and 1 mg/mL Hoechst 33342. Fluorescent images of vasopermeation were collected at time 0, 15 and 30 minutes after injection at two different magnifications. A total of 360 still epifluorescence images were collected, each using the same exposure time and magnification. Therefore, each image is comparable with the rest. Fluorescence images were aligned, registered and overlaid to create three color composite images at each time point. Images were not adjusted for contrast or brightness. A total of 66 embryos were utilized for these experiments.

Image capture and processing was performed as discussed in^1^ and used an upright epifluorescence microscope with a motorized Z stage (AxioImager Z1, Carl Zeiss, Thornwood, NY) controlled by Volocity software (Improvision, Lexington, MA).

### Potency Binding

The antigenic target for our antibodies was coated on to microtiter plates and any exposed areas in the wells were coated with blocking buffer consisting of 2% bovine serum albumin (BSA) in 20 mM Tris-HCl, 150 mM NaCl, pH 7.4 (TBS). The PVL samples were serially diluted from 2.5 μg/mL down to 9.75 ng/mL in blocking buffer and compared to identically diluted PVL1 controls. Dilutions of sample and positive control antibodies occurred in triplicate (negative control antibodies in duplicate) and incubation occurred for 1.5 h at 37 °C. After incubation, excess antibody was washed away by rinsing the wells three times with 0.1% Tween 20 in TBS (TBST) and then adding goat anti-human lambda light chain conjugated to horseradish peroxidase (1:1000 dilution; Novus Biologicals). After a 30 min incubation at 37 °C, excess secondary antibody was washed away by rinsing the wells three times with TBST, followed by a 5 min rinse in TBS alone. Colorimetric signal was generated in each well by adding 100 μL of 3,3′,5,5′-tetra-methylbenzidine (TMB Microwell Peroxidase Substrate, KPL, Gaithersburg, MD). Colorimetric development was stopped 3 minutes later by the addition of TMB Stop Solution (also KPL) and read in a SpectraMax M5 Microplate Reader (Molecular Devices, Sunnyvale, CA) at 450 nm. The signal generated at each concentration was plotted as a sigmoidal dose-response curve and the linear portion of the curves from the sample and control were compared. Analysis of variance (ANOVA) was used to determine the relative potency of the sample to the control and this value is presented as a percentage of potency.

### *In Silico* Analysis of PEP Structure

Three-dimensional structure building and all molecular modeling simulations were performed using the SYBYL software suite (Tripos/Certara, St. Louis, MO). Molecular structures were constructed using *Biopolymer.* Energy calculations were performed using the Tripos force field with a distance-dependent dielectric and partial atomic charges calculated using the *Gasteiger-Hückel* method. For energy minimization, the Powell conjugate gradient algorithm was used with a convergence criterion 0.005 kcal/(mol·Å). To explore the conformational space and folding behavior of the peptides, simulated annealing in 50 cycles was performed using the SYBYL *Dynamics* module by heating up to 700 K, then annealing to 0 K and full minimization after each cycle. The separating surfaces between peptide and receptors were rendered by the MOLCAD module.

### *Ex Ovo* detection of vascular leak

A modified Miles assay^2^ was adapted for the CAM model. Chicken embryos (day 15) were injected intravenously with phosphate buffered saline (PBS), H1-N, PEP, IL-2 or one of the PVL constructs in 50 μL volumes. For local applications, reagents were applied to the CAM via a small hole in a sterilized glass coverslip (18 mm diameter). Embryos were then incubated for 2 hours at which time 100 μL of 0.5% Evan’s Blue, 5% BSA in PBS was injected and embryos were further incubated for 60 minutes. After incubation, the embryos were perfused with saline. 1 cm^2^ of tissue underlying the coverslip was surgically excised after the treatment period and blotted dry, weighed, homogenized and incubated in 200 μL of 100% formamide to release the extravasated dye. Tissue samples were homogenized for 30 sec and then incubated for 48 hr at 37 °C. The samples were centrifuged (14000 g for 10 minutes) and 175 μL of supernatant quantified spectrophotometrically against a formamide blank at 620 nm. Vascular permeability index was calculated as dye concentration in treated tissue sections/dye concentration in matched vehicle (PBS) treated samples.

### *In Vivo* detection of vascular leak

A modified Miles assay^2^ was adapted for the rat ear studies as well. Evans Blue dye solution (10 ml/kg body weight, 0.5% Evans blue (w/v) in endotoxin-free PBS) was injected intravenously. Ten minutes after injection, each rat was injected intradermally with 25 μL of PBS into the left ear and 0.15 nmoles of reagent (~25 μL) into the right ear. Thirty minutes later, rats were anesthetized, their ears photographed, and then perfused with 100 mL PBS thru a ventricular infusion to remove free intravascular dye. The ears were removed, the area of extravasation cut out with a biopsy punch (8 mm wide), and then the tissue was weighed and subsequently placed into 1 mL of formamide for elution of the Evans Blue dye at 60 °C over the course of 48 hours. The amount of extravasated dye was then determined spectrophotometrically against a formamide blank at 620 nm. The absolute amount of dye was determined using a standard curve.

## Additional Information

**How to cite this article**: Pink, D. *et al.* High efficacy vasopermeability drug candidates identified by screening in an *ex ovo* chorioallantoic membrane model. *Sci. Rep.* **5**, 15756; doi: 10.1038/srep15756 (2015).

## Supplementary Material

Supplementary Information

## Figures and Tables

**Table 1:** Characteristics of VEA candidates tested with CAMs.

Reagent	Description	Binding Potency	Mol. Wt.*(Daltons)	pI*
PVL 1	**Control:** NHS76 antibody with an IgG2 constant region. No IL-2 fused to antibody.	100%	142,795.7	8.01
PVL 2	Fusion of wildtype, mature IL-2 (133 amino acids) to the carboxy tail of NHS76 (IgG2).	78.5%	173,595.7	8.48
PVL 5	Fusion of IL-2 residues 1–72 (includes full B-helix) with C-tail of NHS76 (IgG2). Point mutation of lysines 8 and 9 into alanines. Point mutation of cysteine 58 into valine.	160.2%	159,491.4	8
PVL 6	Fusion of IL-2 residues 1–57 (includes partial B-helix corresponding to PEP fragment) to C-tail of NHS76 (IgG2). Point mutation of lysines 8 and 9 into alanines.	78.4%	155,851.2	8.48
PVL 9	Fusion of IL-2 residues 1–51 (does not include B-helix) to C-tail of NHS76 (IgG2). Mutation of lysines 8 and 9 into alanines.	Lot 1:166.6% Lot 2:141.1%	154,353.4	8.48
PVL 10	Fusion of IL-2 residues 22–57 (PEP fragment) to C-tail of NHS76 (IgG2). A GGGGSGGGG linker was included between the NHS76 and the PEP fragment.	90.6%	151,610.4	8.63

^*^Calculated from the Expasy website. www.expasy.org.

**Table 2:** Toxicity Screen for VEA constructs. Percent survival of chick embryos.

Reagent	n	24hours	48hours	72hours	Endotoxin(EU / mL)
PBS	**40**	100	100	87.5	0
LPS	**5**	0	0	0	1000
PVL 1	**5**	100	100	80	Lot 1: 3.14
PVL 1	**5**	100	100	100	Lot 2: 1.70
PVL 2	**5**	100	80	80	<0.5
PVL 5	**5**	100	80	80	4.54
PVL 6	**5**	100	100	80	Lot 1: 6.91; Lot 2: 1.40*
PVL 9	**5**	100	100	80	Lot 1: 7.86; Lot 2: 0.89*
PVL 10	**5**	100	100	100	6.58

^*^Two lots of PVL6 and PVL9 were used for the vasopermeation studies, however, only the lot with the higher EU/mL (Lot 1 in each case) was used for the toxicity screen.
