# Supplementary material for: High efficacy vasopermeability drug candidates identified by screening in an ex ovo chorioallantoic membrane model
Source: Sci Rep. 2015 Oct 29;5:15756. doi: 10.1038/srep15756 (PMC4625159; doi:10.1038/srep15756)

High efficacy vasopermeability drug candidates identified by screening in an *ex ovo* chorioallantoic membrane model.

Desmond Pink[2,4], Keith A. Luhrs[1,9], Longen Zhou[6,9], Wendy Schulte[2], Jennifer Chase[2], Christian Frosch[7], Udo Haberl[7], Van Nguyen[8], Aparna I. Roy[9], John D. Lewis[2,4], Andries Zijlstra[2,3*], Missag H. Parseghian[5,9*]

[1]Allergan Inc., 2525 Dupont Dr., Irvine, CA, 92612, USA;
[2]Innovascreen Inc., 1959 Upper Water St. Suite 1700, Halifax, NS, B3J 3N2, Canada;
[3]Vanderbilt University Medical Center, 1161 21st Ave. S., Nashville, TN, 37232, USA;
[4]University of Alberta, 114th Street & 87th Avenue, Edmonton, AB, T6G 2E1, Canada;
[5]Rubicon Biotechnology, 26212 Dimension Drive, Suite 260, Lake Forest, CA, 92630, USA;
[6] NantBioScience, 101 Theory Street, Suite 150, Irvine, CA 92617, USA;
[7]BioBroker, Arnold-Sommerfeld-Ring 2, 52499 Baesweiler, Germany;
[8]Currently, Peregrine Pharmaceuticals Inc., 14282 Franklin Avenue,
Tustin, CA, 92780, USA;
[9]Formerly, Peregrine Pharmaceuticals Inc.

*** To whom all correspondence should be addressed**

Missag H. Parseghian, PhD
Chief Scientific Officer
Rubicon Biotechnology
26212 Dimension Drive, Suite 260, Lake Forest, CA 92630 USA

Former Address:
Peregrine Pharmaceuticals Inc.,
14272 Franklin Avenue, Tustin, CA, USA;
Phone: 1-818-422-5734
email: missaghp@earthlink.net

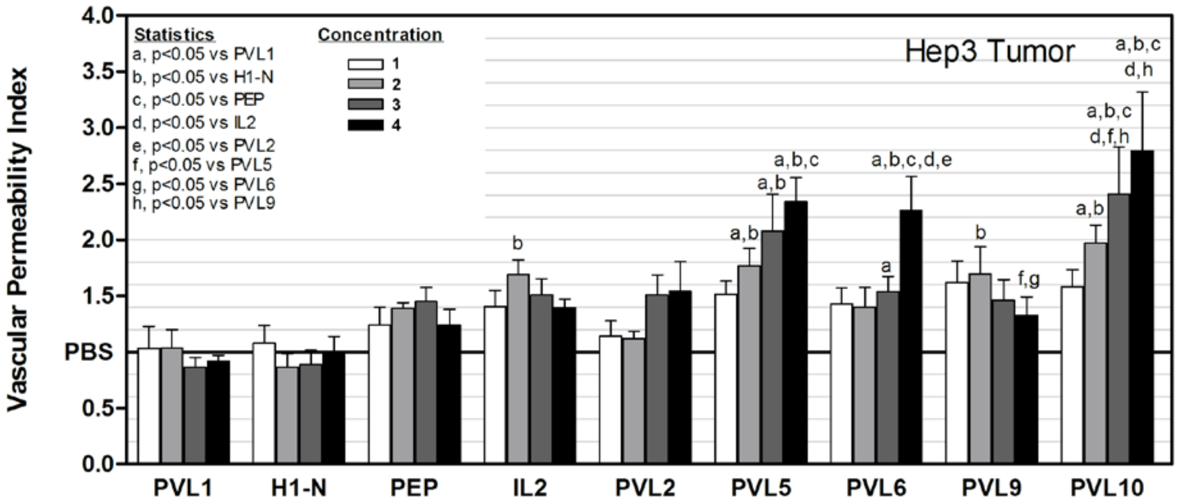

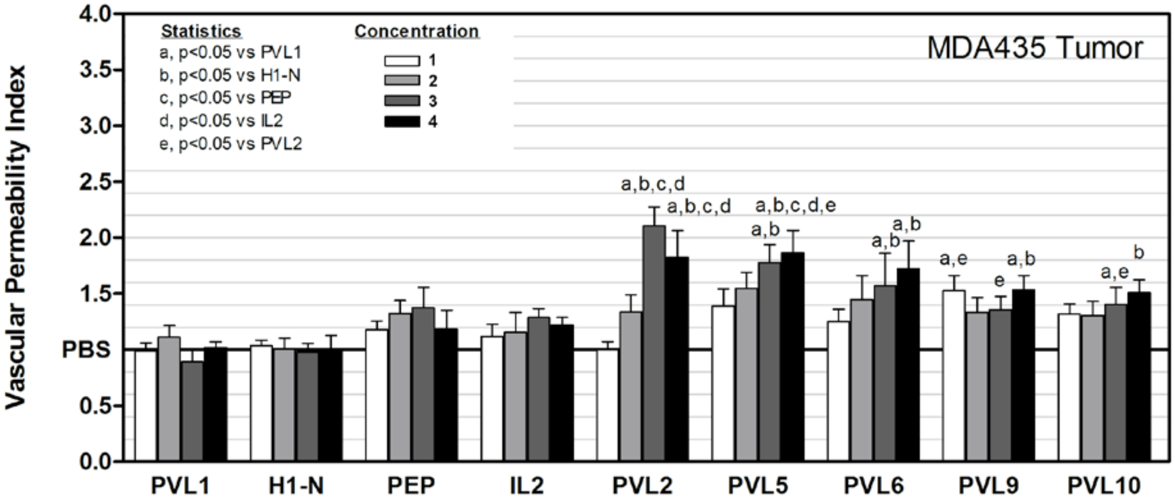

Supplementary Figure 1. Vascular permeability indices in chick embryos after intravenous injection of control and PVL reagents in HEp3 bearing (top graph) and MDA-MB-435 bearing (bottom graph) CAMs. Average VPIs and standard errors determined for each PVL candidate were obtained from 3 experiments run in triplicate (n=9) at each concentration, which were as follows: Concentrations for control reagents are (1) 0.001 nM, (2) 0.01 nM, (3) 0.1 nM and (4) 1 nM; Concentrations for experimental reagents are (1) 0.005 nM, (2) 0.05 nM, (3) 0.1 nM and (4) 0.15 nM.

Supplementary Information (following pages). Sample images for each PVL candidate tested in MDA-MB-435 and HEp3 bearing CAMs are provided in 14 slides. Rhodamine-LCA staining of the vasculature in HEp3 tumors reveals a strong and consistent signal suggesting greater vascularization in that tumor microenvironment compared to the MDA-MB-435 tumors. Images were captured as described in (Pink et al., 2012).

PBS: Hep3 tumor 9.4x magnification

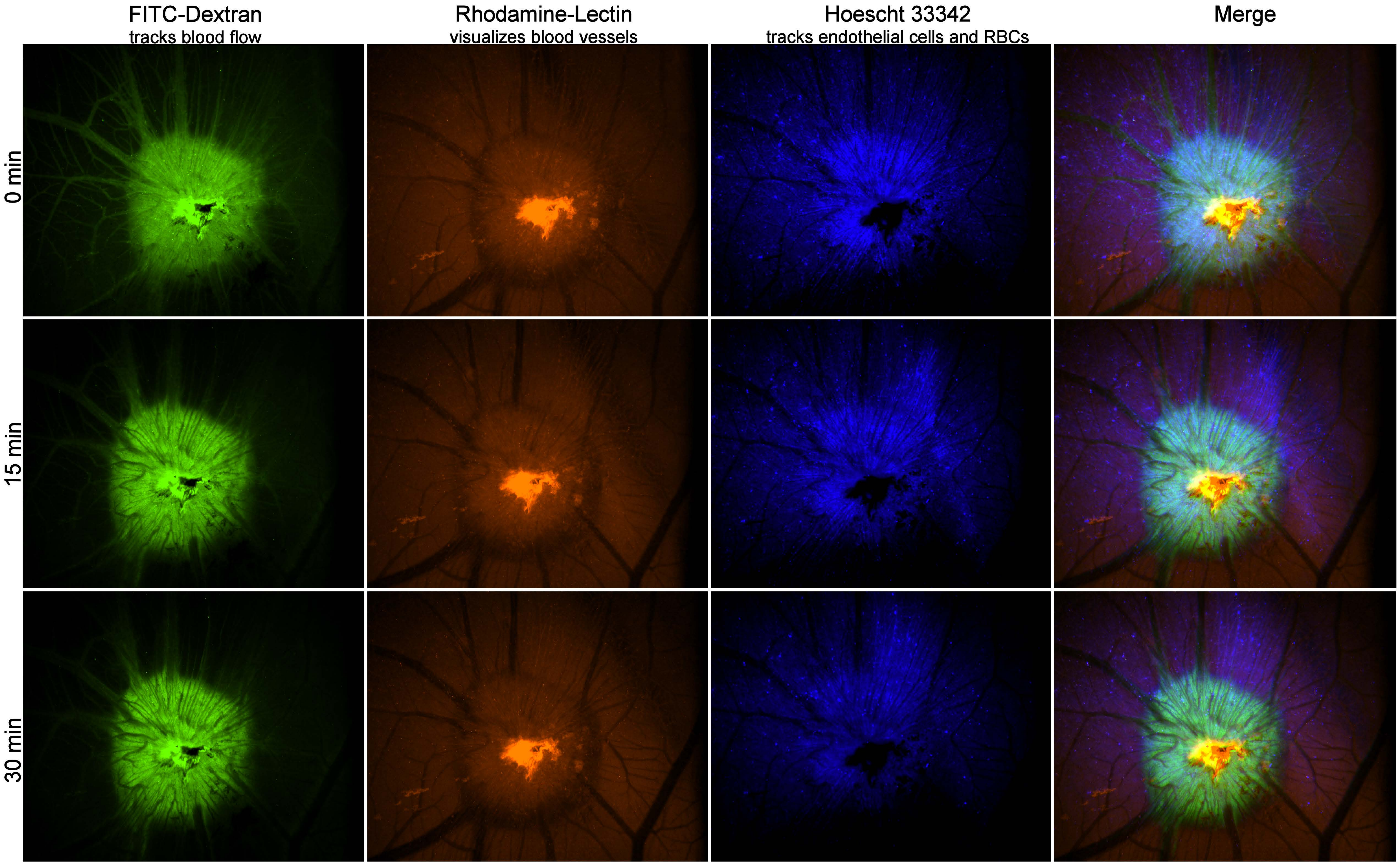

PVL1: Hep3 tumor 13.2x magnification

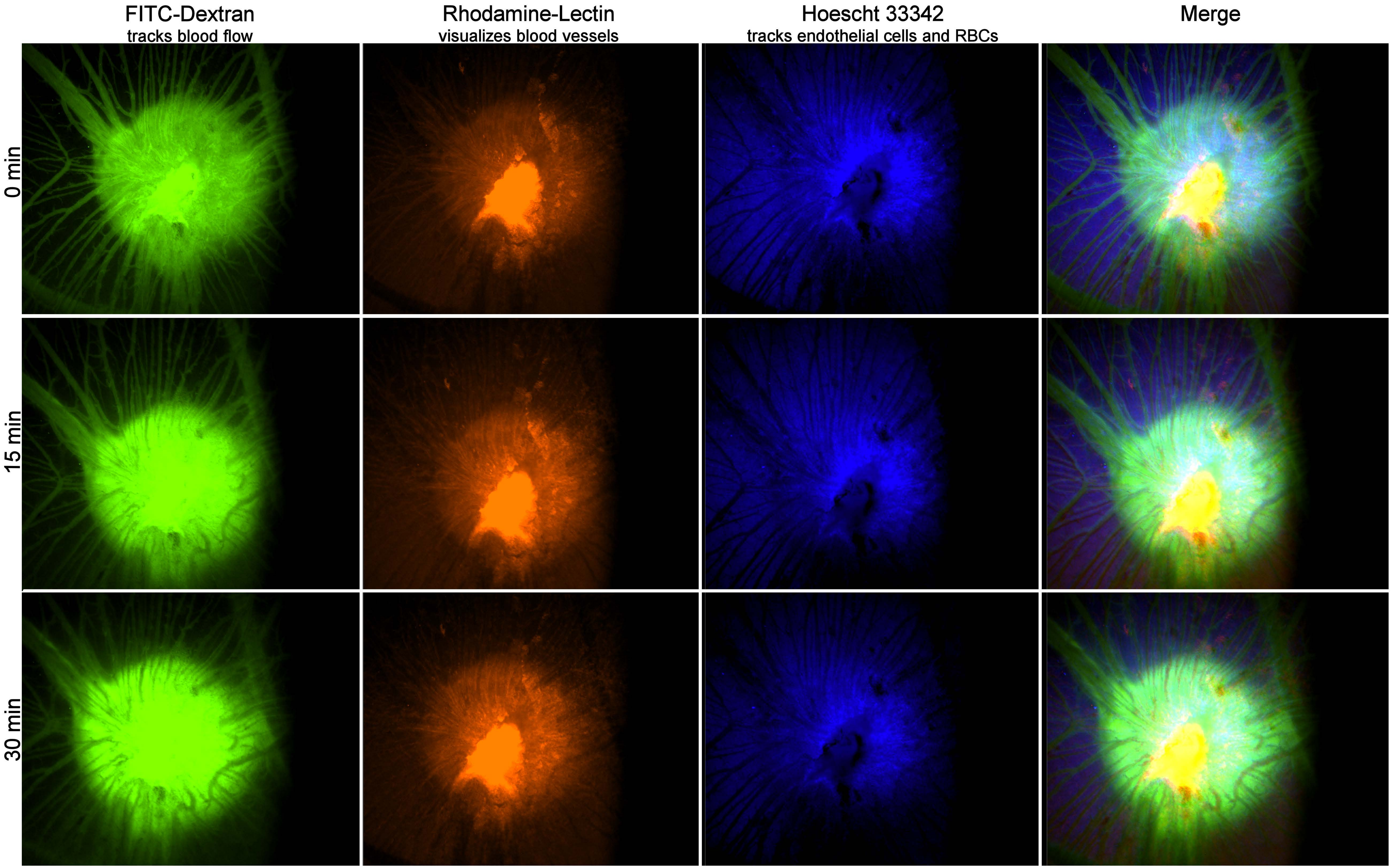

PVL2: He3 tumor 6.4x magnification

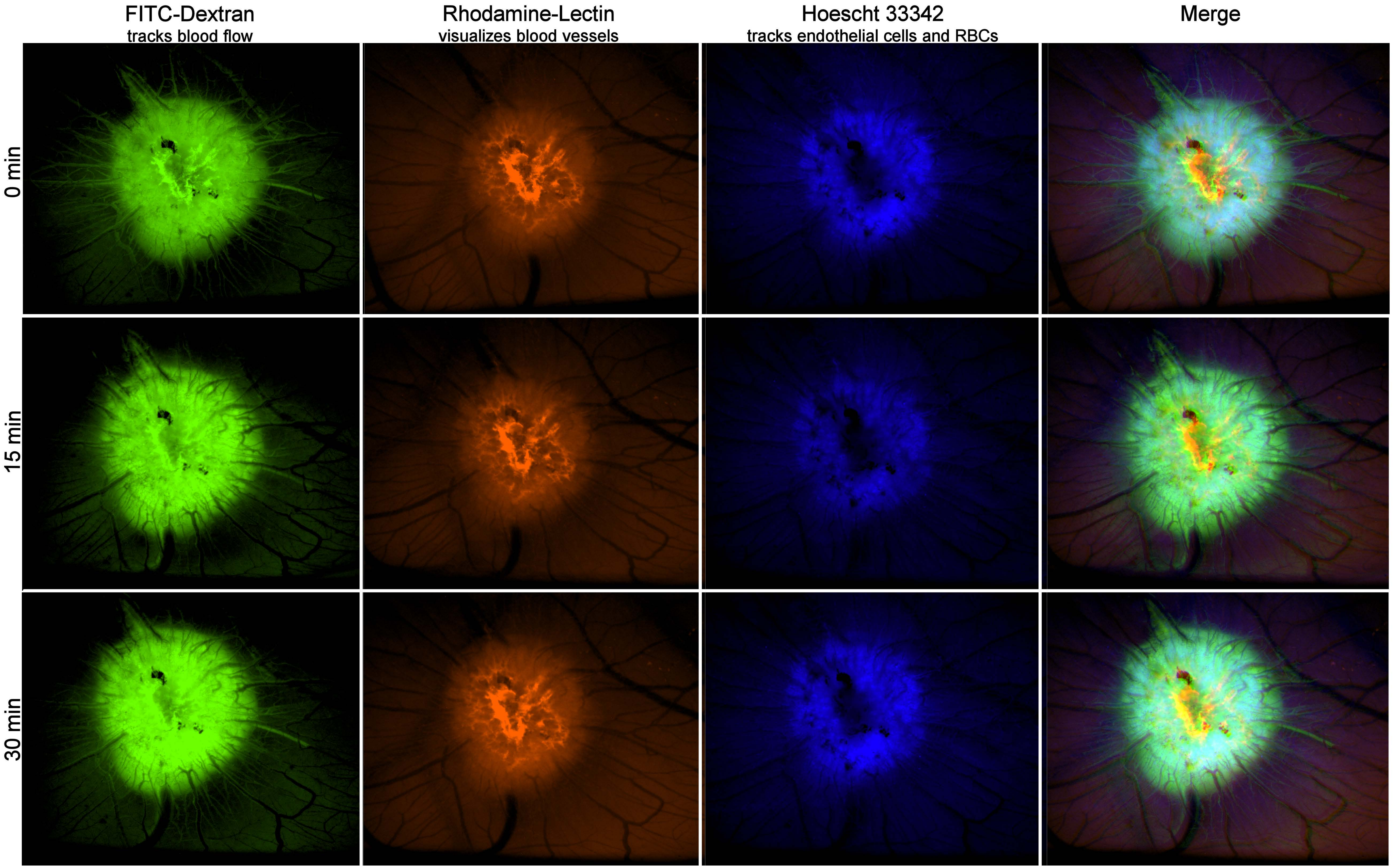

PVL5: Hep3 tumor 10.1x magnification

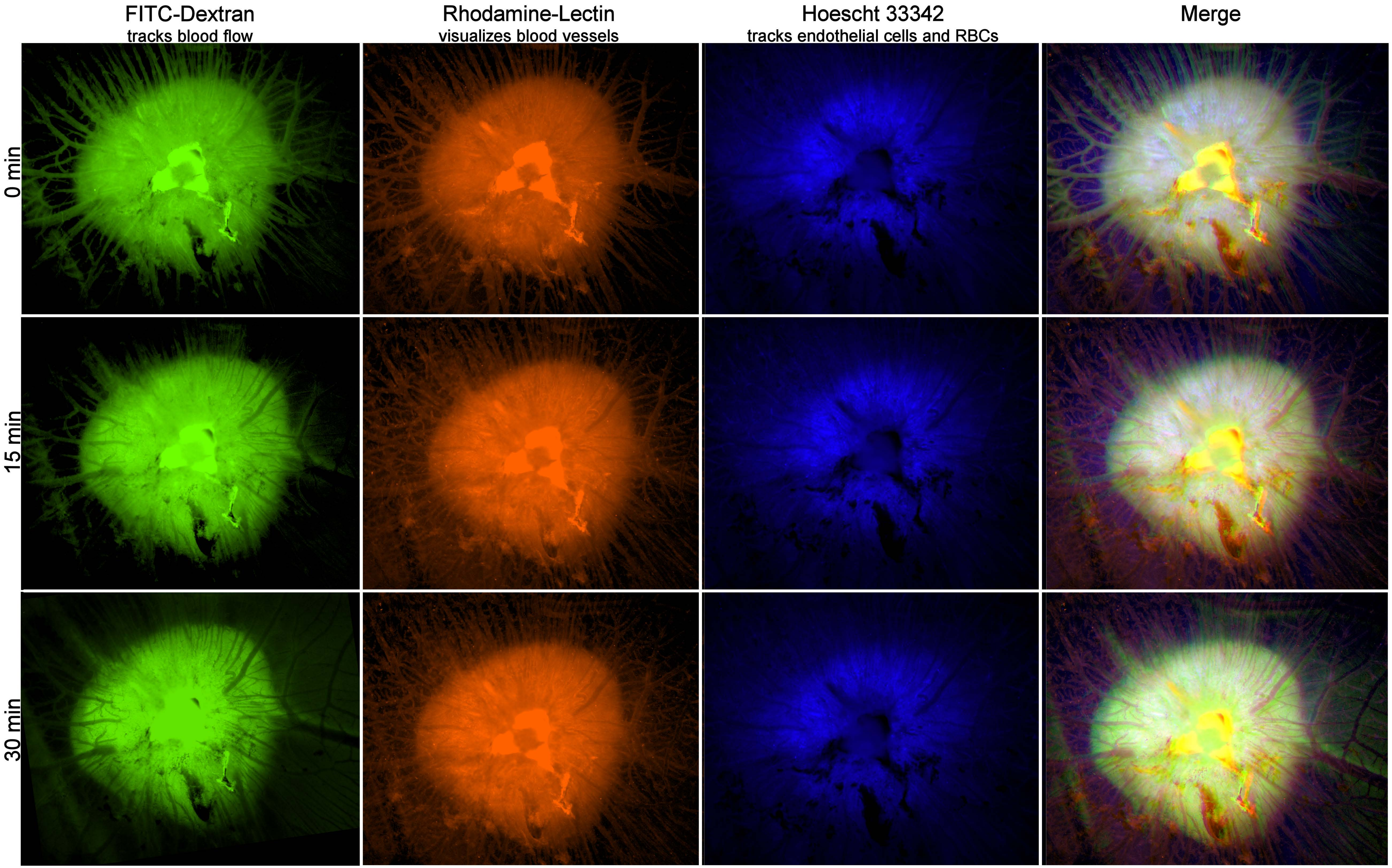

PVL6: Hep3 tumor 8.0x magnification

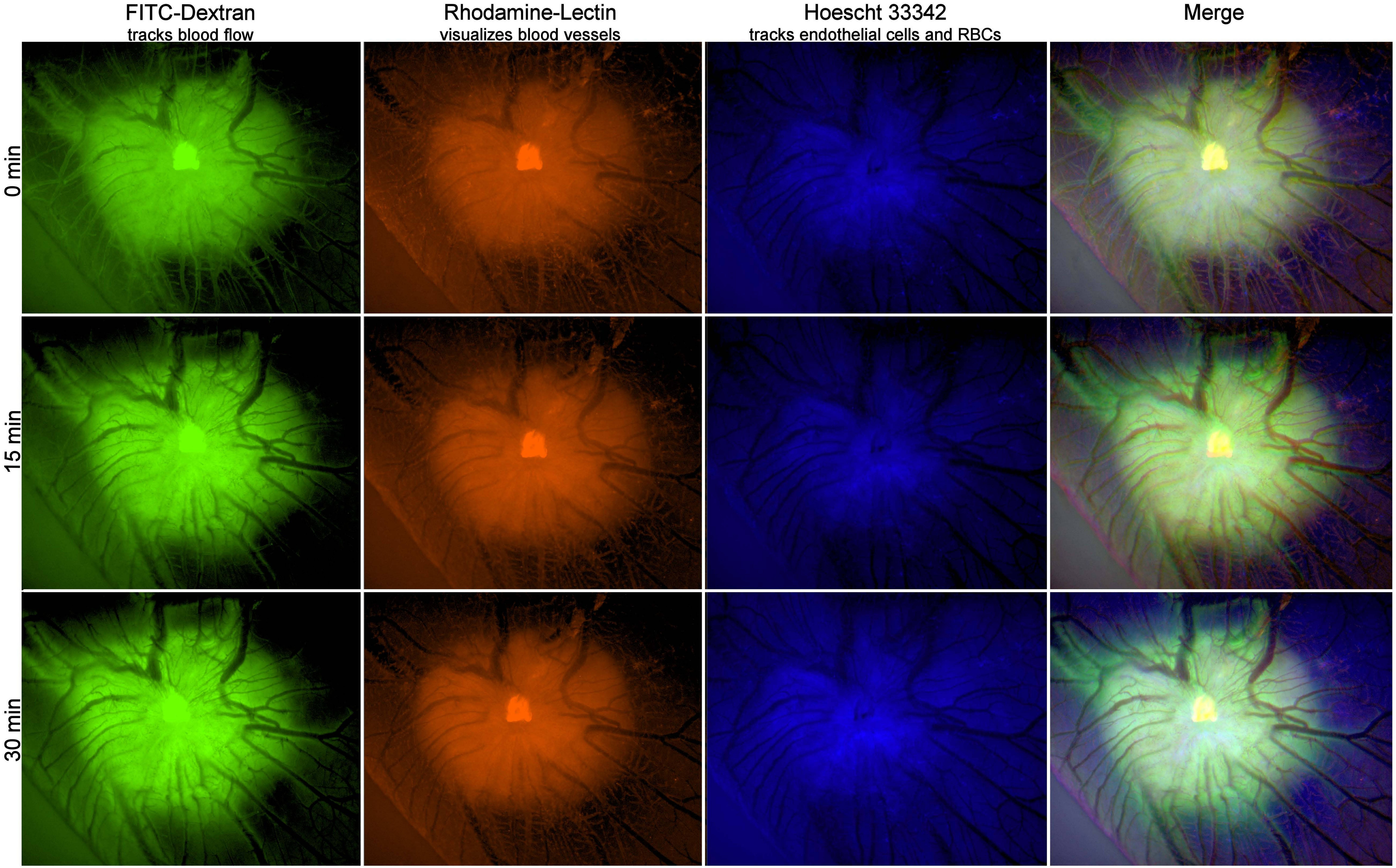

PVL9: Hep3 tumor 15.6x magnification

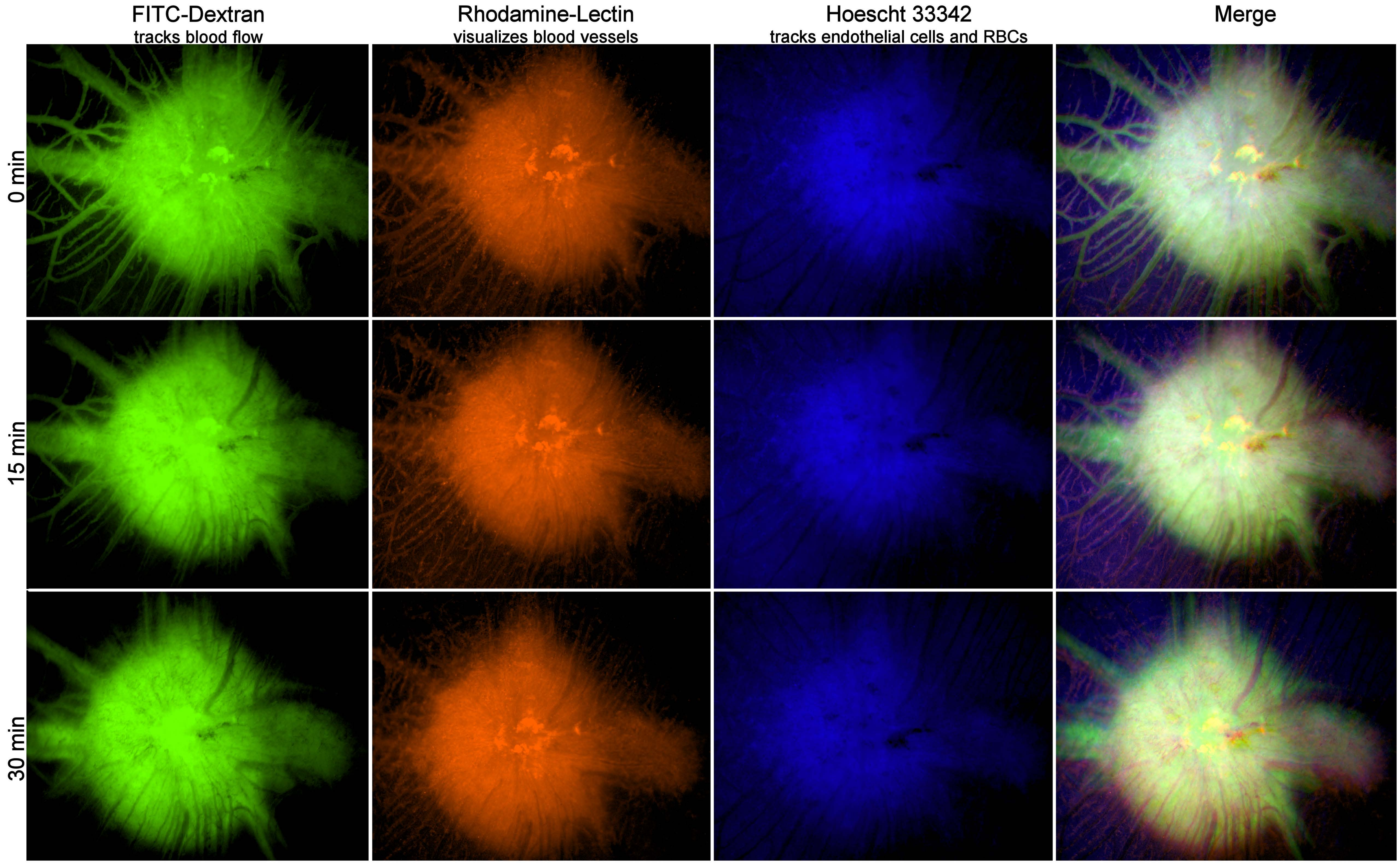

PVL10: Hep3 tumor 11.2x magnification

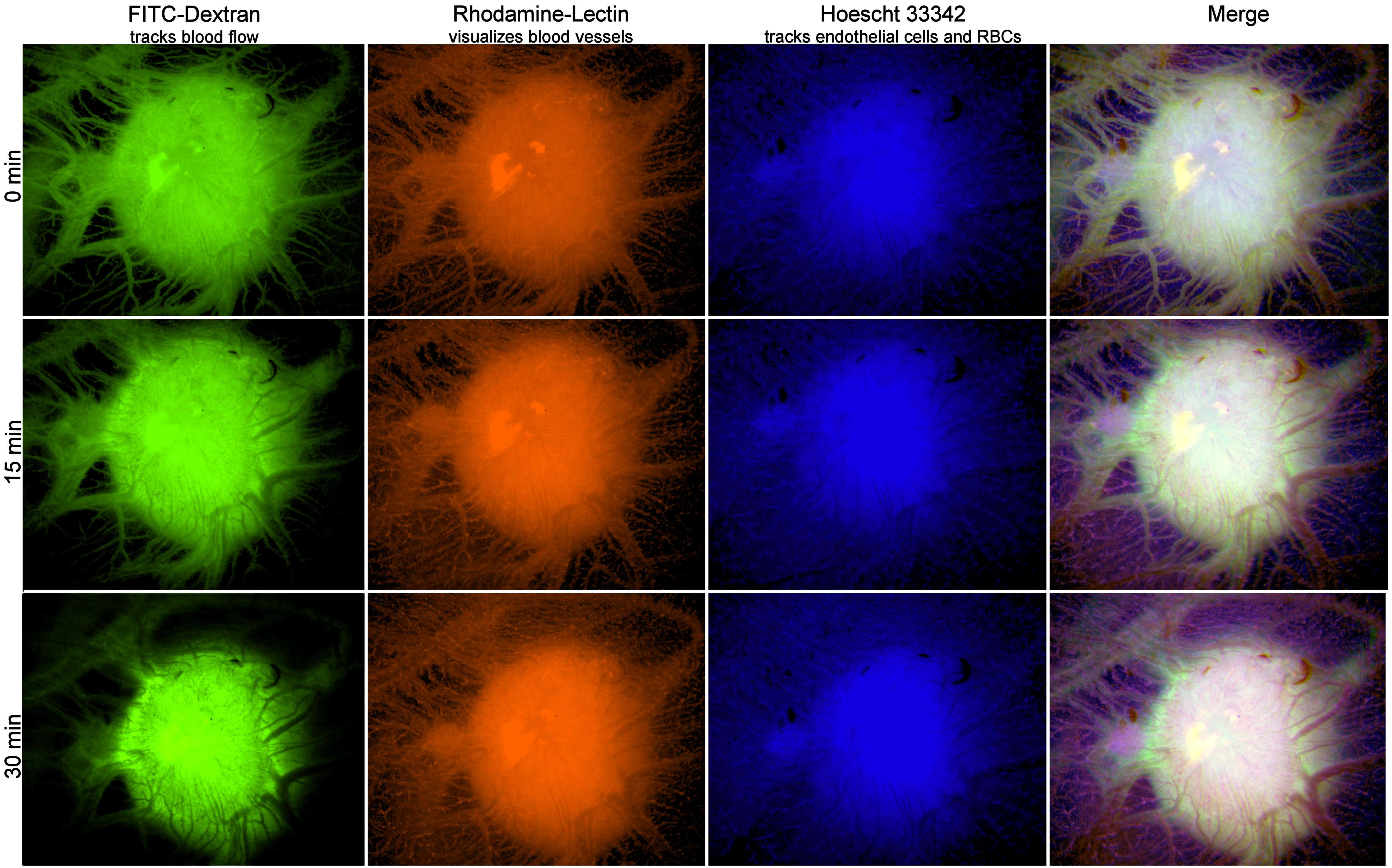

PBS: MDA-MB-435 tumor 16.2x magnification

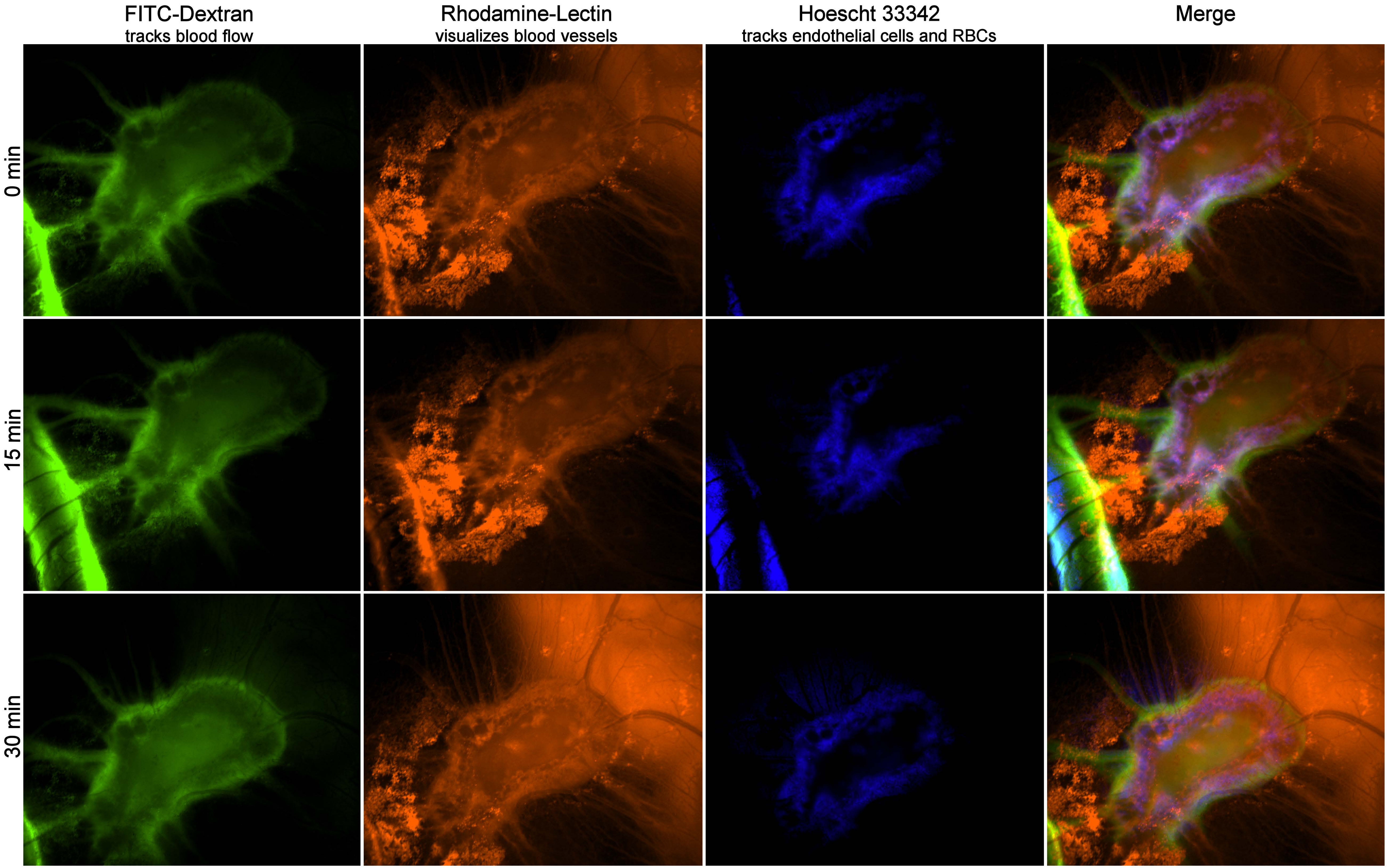

PVL1: MDA-MB-435 tumor 16.2x magnification

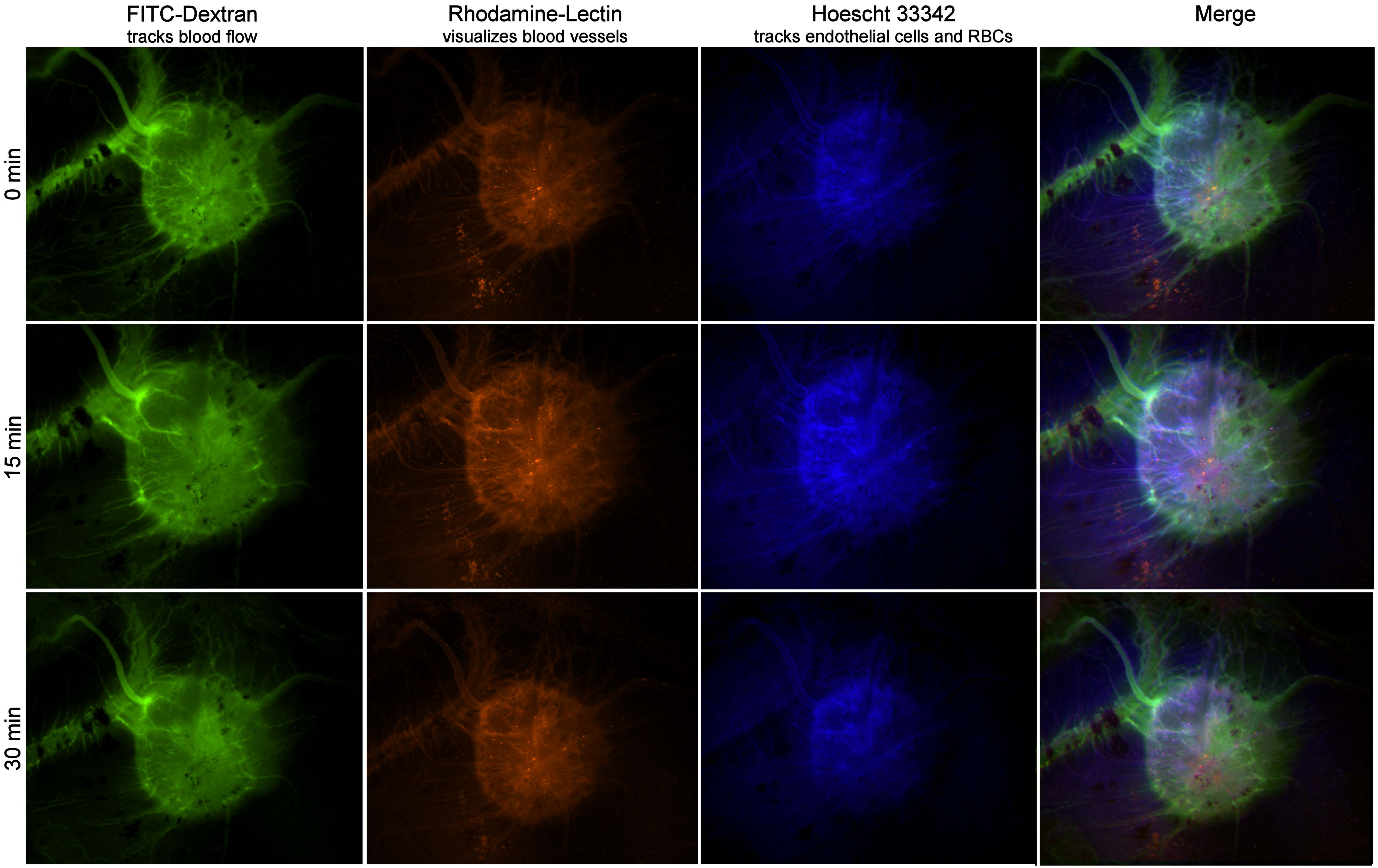

PVL2: MDA-MB-435 tumor 16.2x magnification

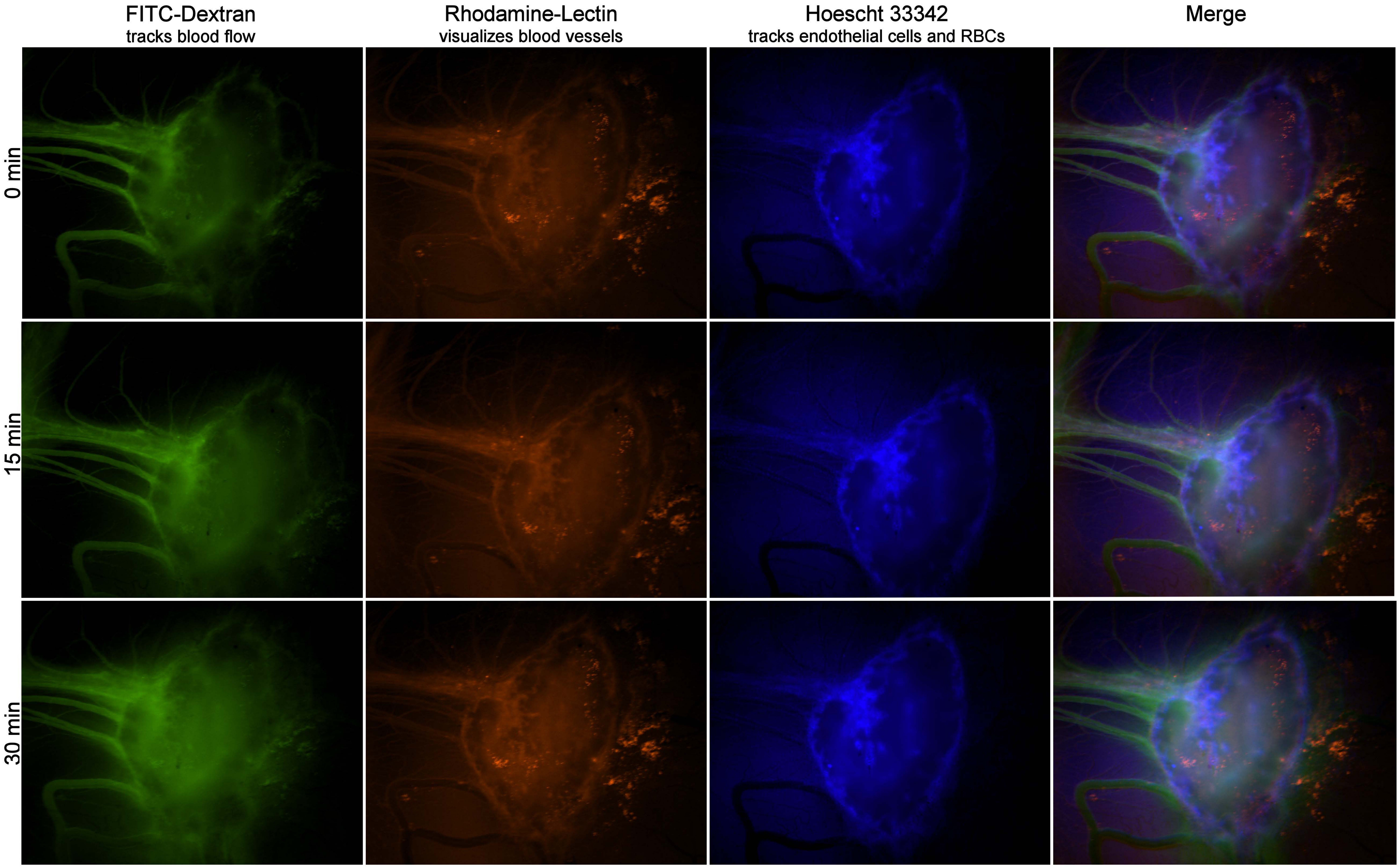

PVL5: MDA-MB-435 tumor 11.7x magnification

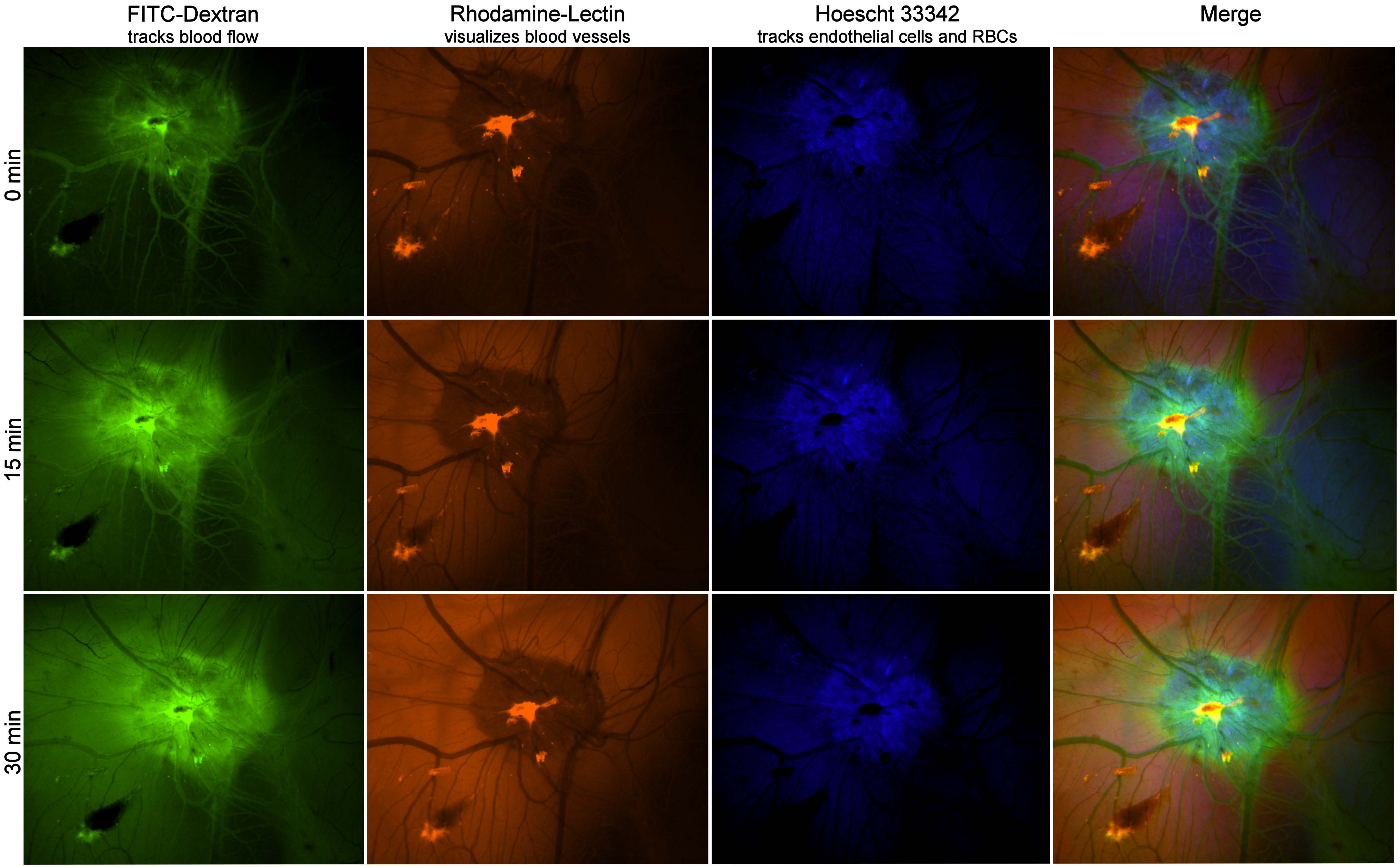

PVL6: MDA-MB-435 tumor 13.6x magnification

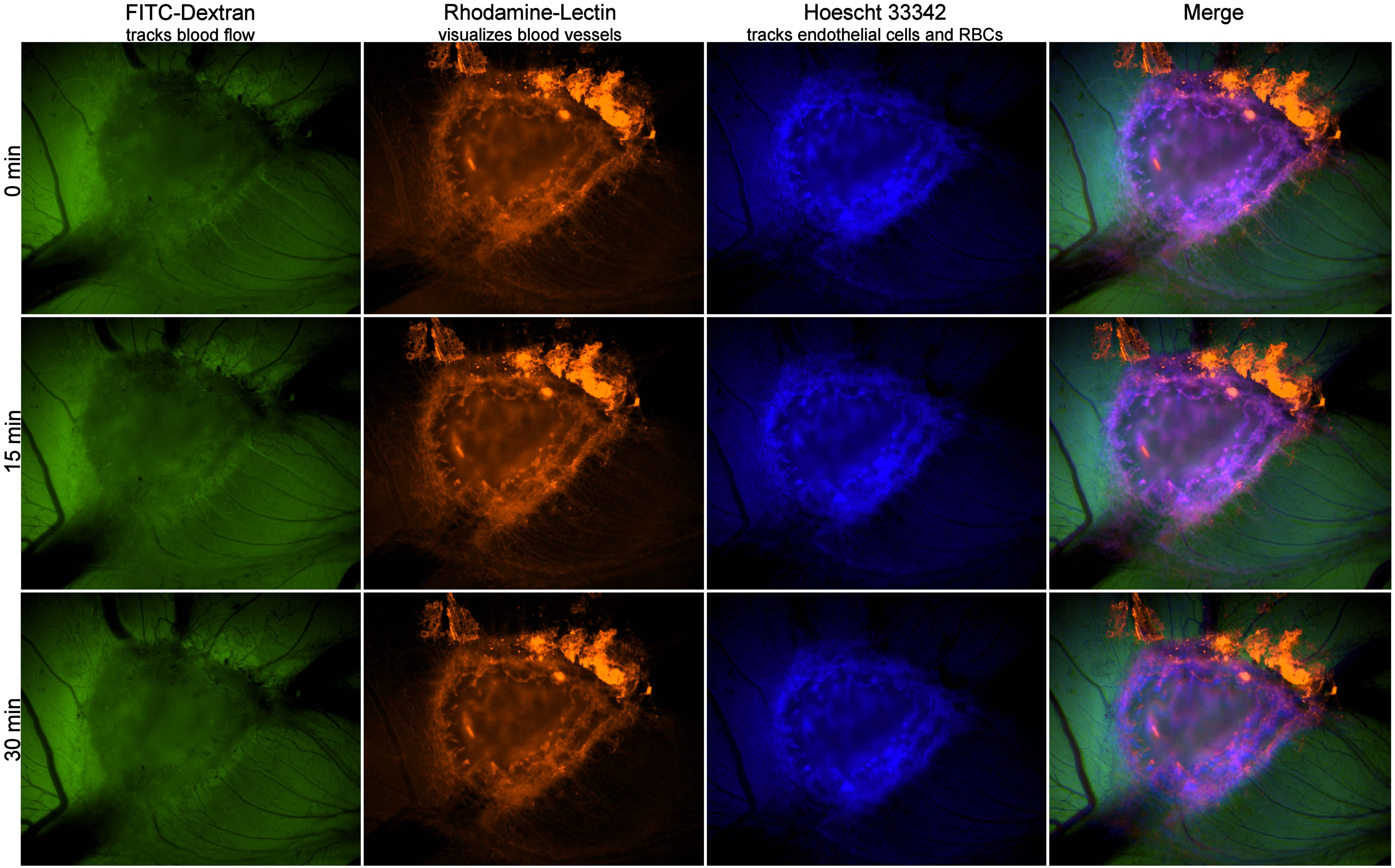

PVL9: MDA-MB-435 tumor 12.5x magnification

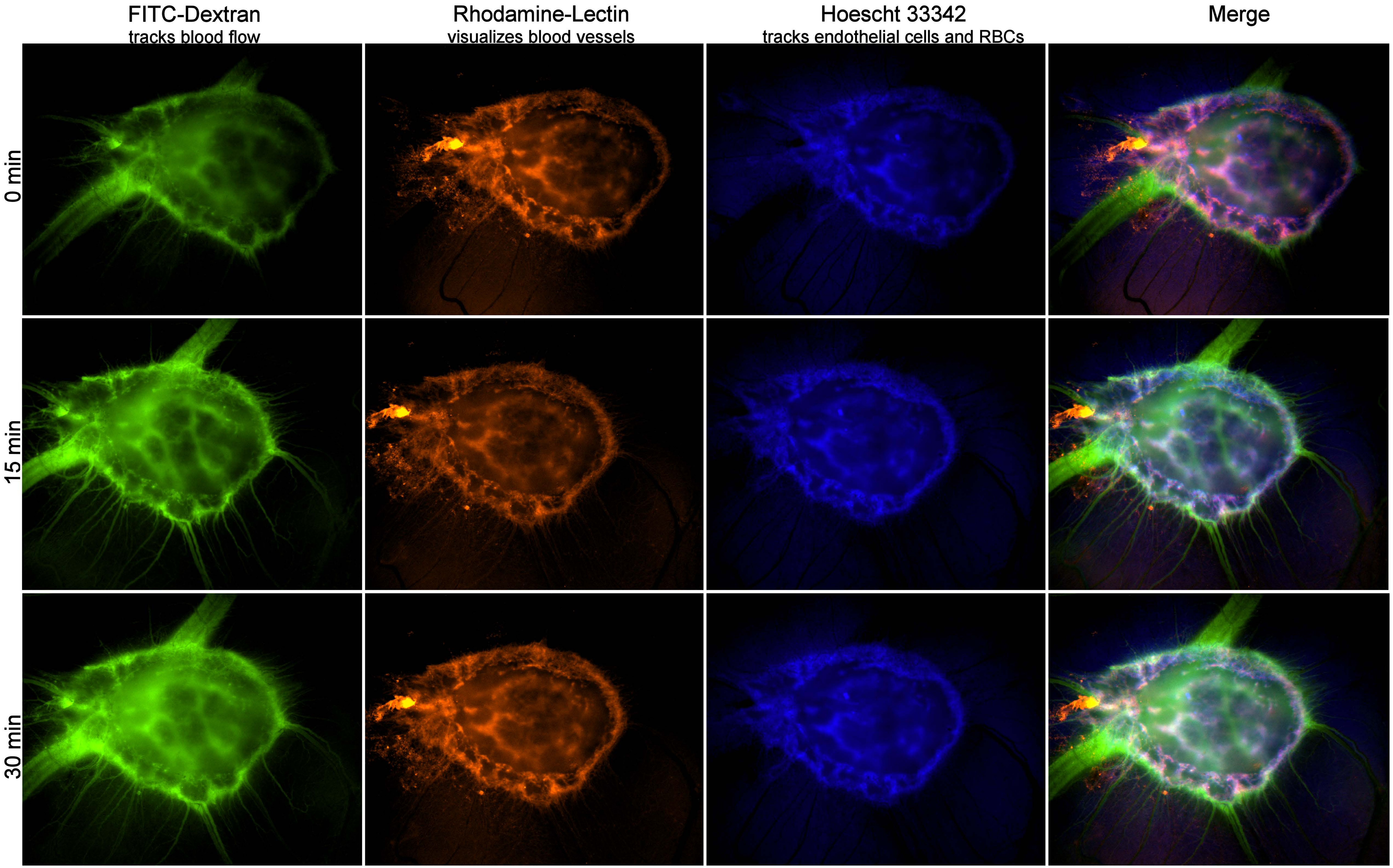

PVL10: MDA-MB-435 tumor 17.8x magnification

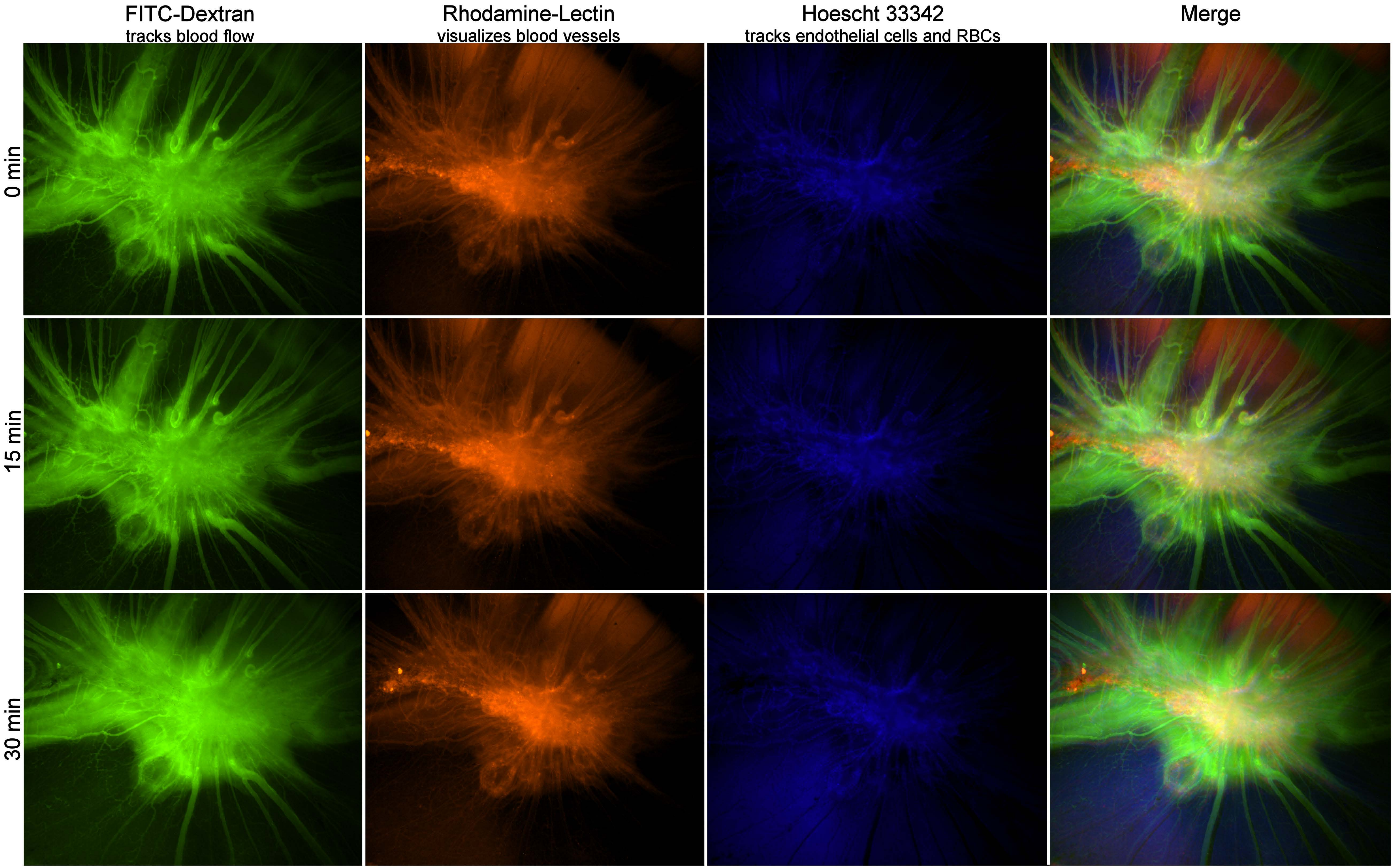

Supplement: Supplementary Information [file srep15756-s1.pdf]
